# CRISPR/Cas9-compatible plasmids enabling seven dominant genetic selection methods for the human fungal pathogen *Cryptococcus neoformans*

**DOI:** 10.1128/spectrum.01935-25

**Published:** 2025-09-25

**Authors:** Michael J. Boucher, Hiten D. Madhani

**Affiliations:** 1 Department of Biochemistry and Biophysics, University of California 8785, San Francisco, California, USA; Jawaharlal Nehru Centre for Advanced Scientific Research, Bangalore, India

**Keywords:** *Cryptococcus neoformans*, markers, CRISPR/Cas9

## Abstract

*Cryptococcus neoformans* is the most common cause of human fungal meningitis and an important model system for studying fundamental eukaryotic biology. Genetic manipulation of this organism relies on three dominant drug resistance markers (nourseothricin acetyltransferase [*NAT*], neomycin phosphotransferase II [*NEO*], and hygromycin B phosphotransferase [*HYG*]) and the recyclable dominant prototrophic marker *amdS*. With ongoing technological advances that are expanding our ability to explore cryptococcal gene function, contemporary studies often require multiple genetic manipulations in the same strain. Additional dominant selection methods would maximize the utility of these tools by facilitating their combinatorial use. Here, we identify blasticidin S resistance via the blasticidin S deaminase (*BSD*) or blasticidin S resistance (*BSR*) markers as a novel dominant selection method for *C. neoformans*. We further validate phleomycin resistance via the bleomycin resistance gene (*BLE*) marker as an additional selection method, confirming a study that first established this marker 25 years ago (J. Hua, J. D. Meyer, and J. K. Lodge, Clin Diagn Lab Immunol 7:125–128, 2000, https://doi.org/10.1128/cdli.7.1.125-128.2000). To enable highly efficient CRISPR/Cas9-mediated genome modification, we incorporated these markers, as well as the newly established dominant prototrophic marker *ptxD* (M. Khongthongdam, T. Phetruen, and S. Chanarat, Microbiol Spectr 13:e01618-24, 2025, https://doi.org/10.1128/spectrum.01618-24), into a vector series that enables the construction of fused marker-sgRNA products via PCR. Altogether, this work expands the number of dominant genetic selection methods for *C. neoformans* to seven, including five drug selection regimes and two prototrophic methods. The vector series has been deposited at Addgene.

**IMPORTANCE:**

*Cryptococcus neoformans* is the top-ranked World Health Organization priority fungal pathogen due to its widespread distribution and inadequate treatment options. Additionally, as a basidiomycete yeast occupying an underexplored branch of the fungal kingdom, this organism is a powerful system for deciphering core eukaryotic biology that is absent in classic model fungi. Defining functions for novel cryptococcal genes is a crucial priority, and the availability of additional genetic selection methods would facilitate these efforts. In this study, we establish blasticidin S resistance as a novel genetic selection method for *C. neoformans*, and we validate a previous report using phleomycin resistance as such. This work expands the number of reliable dominant selection methods to seven, providing flexibility for the introduction of sequential genetic modifications into single strains.

## OBSERVATION

*Cryptococcus neoformans* is an environmental fungus of both basic and biomedical significance (1). As a basidiomycete yeast that diverged from ascomycetes at least 450 million years ago (2), this organism encodes otherwise-conserved eukaryotic pathways that have been lost from traditional model fungi. Facile growth conditions and robust molecular genetics have made it an important model for dissecting such pathways (3–8). Clinically, *C. neoformans* causes opportunistic meningitis in immunocompromised individuals—particularly those with CD4^+^ T-cell deficiencies—leading to >118,000 annual deaths that include 19% of global HIV/AIDS-related mortality (9, 10). Despite its position in the “critical” tier of World Health Organization priority fungal pathogens (11), our understanding of how cryptococcal genes influence its infection biology remains in its infancy.

The haploid genome and defined sexual cycle of *C. neoformans* have made it well-suited for genetic studies. The development of biolistic transformation methods in the 1990s enabled the functional analysis of cryptococcal genes (12), including critical virulence factors. Construction of arrayed gene deletion libraries has driven systematic studies that have begun to build large-scale functional maps of this organism’s genome (8, 13–18). More recently, application of CRISPR/Cas9 tools to this system has streamlined genetic manipulation, replacing cumbersome biolistic protocols with electroporation and reducing the amount of homology necessary for gene replacement to just 50 bp (19–22). Further advances in robust genome-wide transposon mutagenesis (23), improved efficiency and accuracy of genetic modification (24, 25), and conditional knockdown systems (26, 27) are rapidly expanding the scope of genetic questions that can be probed.

 Contemporary genetic manipulation of *C. neoformans* uses dominant markers to select for transformants. Drug resistance markers encoding hygromycin B phosphotransferase (*HYG*), neomycin phosphotransferase II (*NEO*), and nourseothricin acetyltransferase (*NAT*) confer resistance to hygromycin B, G418, and nourseothricin, respectively (28–30), and are the predominant selection methods used in the field. More recently, dominant prototrophic markers have been developed, with a marker encoding the *Aspergillus nidulans* acetamidase (*amdS*) enabling the use of acetamide as a sole nitrogen source (31), and a marker encoding the *Pseudomonas stutzeri* phosphite dehydrogenase (*ptxD*) allowing the use of phosphite as a sole phosphorus source (32). The *amdS* marker is particularly valuable because it confers sensitivity to fluoroacetamide and can therefore be counter-selected, enabling marker recycling or repair of disrupted loci (25, 31).

 As the number of genetic tools in *C. neoformans* expands, the availability of additional selectable markers will facilitate its study. For example, the expression of Cas9 for CRISPR-based genome editing (22), TetR fusions for RNA-level gene regulation (26), or *Os*Tir1 for auxin-dependent protein depletion (27) each requires the use of a marker. Combinations of such tools, along with knockouts and complementation of genes of interest, can quickly consume all available selection methods. While recycling *amdS* is one possible solution for multiple manipulations, this may not be optimal in all situations, such as when it has been used to temporarily delete genes that one may want to subsequently restore (25). Expanding the repertoire of dominant markers will thus enhance the complexity of genetic studies that can be performed in this organism.

Toward this end, we tested whether the commonly used selection drugs puromycin, phleomycin, and blasticidin S inhibit fungal growth on solid agar. We note that phleomycin was originally established for the selection of *C. neoformans* transformants 25 years ago (29) but has not, to our knowledge, been subsequently reported in the literature. While puromycin failed to inhibit fungal growth at concentrations up to 500 µg/mL, phleomycin and blasticidin S completely inhibited growth at 200 and 500 µg/mL, respectively (Fig. S1).

**Fig 1:**
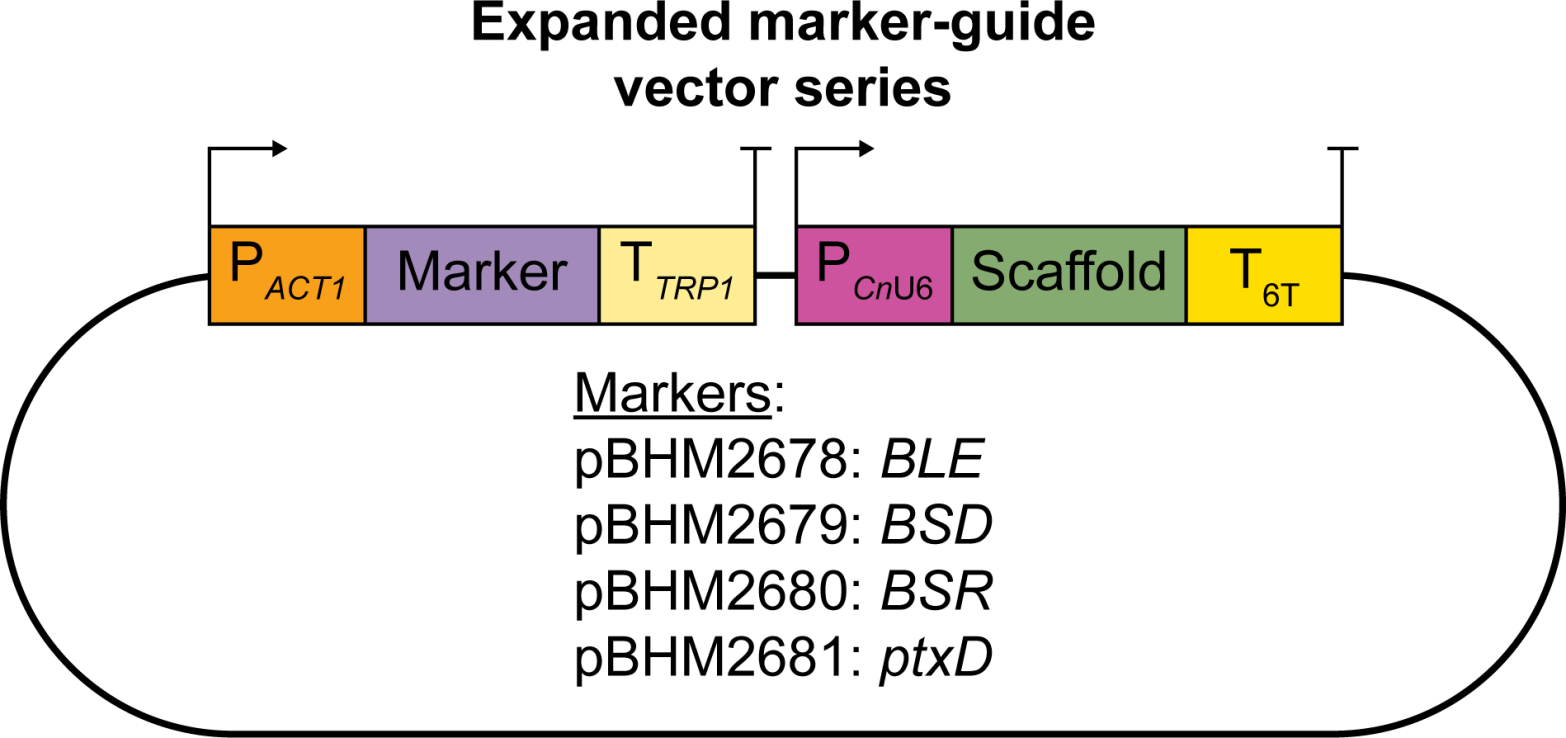
Vector series to produce marker-guide fusions using the *BLE***,** *BSD***,** *BSR***,** or *ptxD* markers.

 In a recent study, we found that electroporation of fused marker-guide PCR products containing overhangs for homology-directed repair (HDR) yielded near-perfect genome-editing efficiency when modifying a parent strain that both expresses Cas9 and lacks the non-homologous end joining (NHEJ) factor Ku80 (25). To determine whether phleomycin and blasticidin S could select for *C. neoformans* transformants, we expanded the marker-guide vector series reported in that work to include markers conferring resistance to phleomycin (bleomycin resistance gene [*BLE]*) and blasticidin S (blasticidin S deaminase [*BSD*] and blasticidin S resistance [*BSR*]) (Fig. 1). We also constructed a vector expressing the newly developed marker *ptxD* (32). In each case, we codon-optimized markers for *C. neoformans* expression and inserted an intron into an arbitrary location approximately one-third of the way through each coding sequence to improve transgene expression (22). As has been done with the most commonly used markers, we drove expression using the *ACT1* promoter and the *TRP1* terminator.

**Fig 2:**
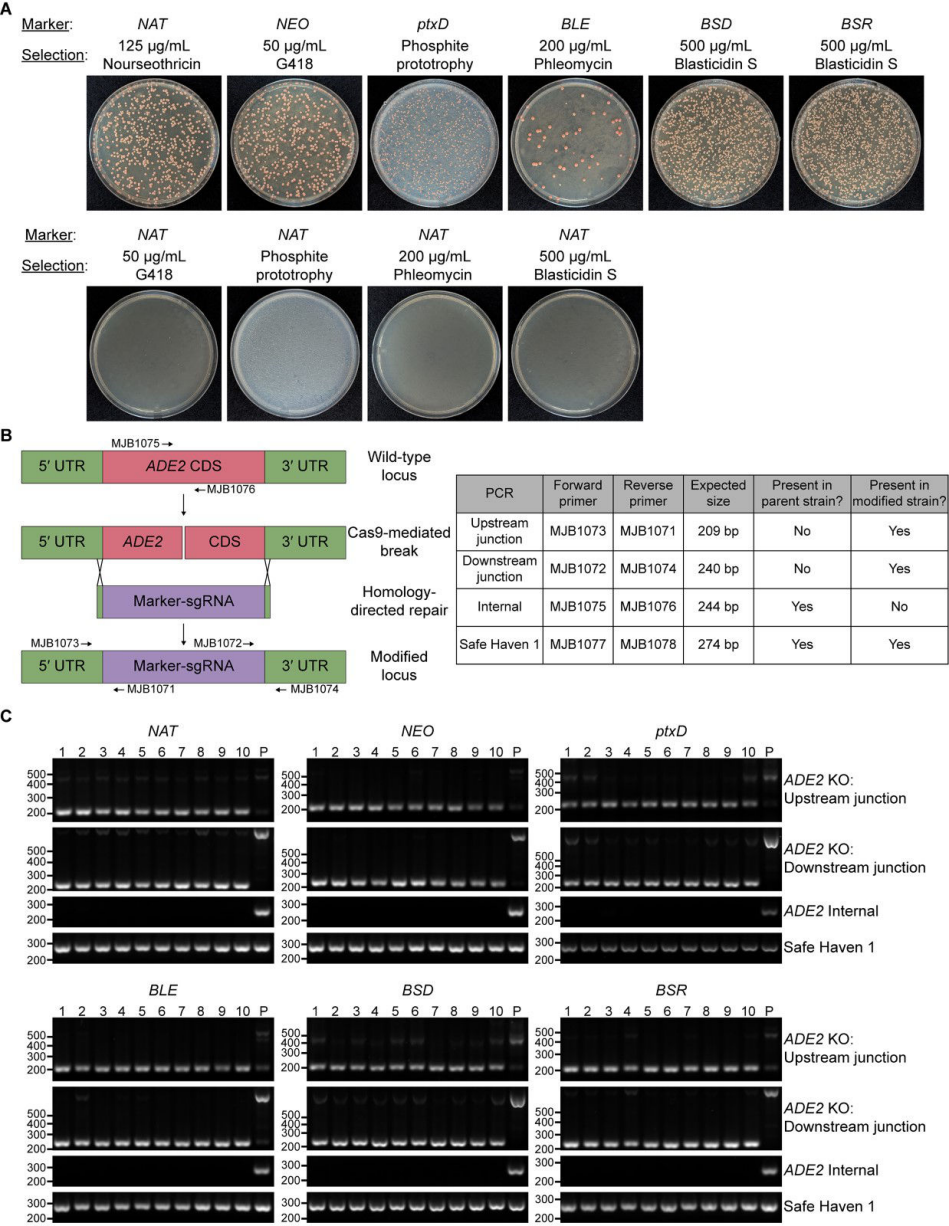
Efficient disruption of *ADE2* using novel and established selectable markers. A Cas9-expressing, *yku80*-blaster strain (CM2465) was electroporated with fused marker-guide constructs as described in the text. (**A**) Electroporated cultures were plated onto selection media corresponding to the appropriate marker (top row) or onto mismatched selection media to assess the frequency of background colonies (bottom row). Plates were incubated at 30°C for 3–4 days followed by storage at 4°C for 3 weeks to allow the red color characteristic of *ade2*Δ cells to emerge. (**B**) Diagram (not to scale) of the expected *ADE2* modification by HDR (left) and table of expected diagnostic PCR results (right). (**C**) Diagnostic PCR assessing transformants for the presence of modified upstream and downstream *ADE2* junctions and the absence of *ADE2* coding sequence. The safe haven 1 locus serves as a positive control for PCR. Numbers above gels indicate transformant clone numbers. *P* indicates the untransformed parental strain.

 For each marker, we electroporated a Cas9-expressing, *yku80*-blaster parent strain with a PCR product containing (i) the marker of interest; (ii) an sgRNA targeting the coding sequence of the *ADE2* gene; and (iii) 50 bp homology arms flanking the *ADE2* coding sequence. All markers yielded transformants when plated onto their corresponding selection plates (Fig. 2A, top, and Table 1). “Mismatched” selection of *NAT* transformants on G418, phosphite, phleomycin, or blasticidin S plates failed to yield colonies (Fig. 2A, bottom), indicating an absence of spurious background colonies. Nearly all colonies developed red pigmentation characteristic of *ADE2* deletion (Fig. 2A and Fig. S2), indicating efficient gene disruption. We confirmed this by isolating genomic DNA from 10 clones transformed with each marker and using PCR to assess (i) disruption of the *ADE2* coding sequence and (ii) HDR at each end of the *ADE2* coding sequence (Fig. 2B). Consistent with our previous work (25), all tested clones (10/10 colonies for all markers) lacked detectable *ADE2* coding sequences and were positive for 5′ and 3′ junction PCR products characteristic of HDR (Fig. 2C).

**TABLE 1:** Transformants obtained in a Cas9-expressing, *yku80*-blaster background

Marker	Selection	Experiment number	Transformants	Percentage of transformants relative to *NAT*
*NAT*	Nourseothricin (125 μg/mL)	1	840	100.0
		2	1,180	100.0
		3	780	100.0
*NEO*	G418 (50 μg/mL)	1	670	79.8
		2	1,310	111.0
		3	1,300	166.7
*ptxD*	Phosphite prototrophy	1	1,560	185.7
		2	1,050	89.0
		3	810	103.8
*BLE*	Phleomycin (200 μg/mL)	1	90	10.7
		2	51	4.4
		3	59	7.5
*BSD*	Blasticidin S (500 μg/mL)	1	3,270	389.3
		2	4,000	339.0
		3	2,530	324.4
*BSR*	Blasticidin S (500 μg/mL)	1	3,290	391.7
		2	4,100	347.5
		3	1,860	238.5

We observed that the *BLE* marker reproducibly yielded fewer transformants compared to other markers (Table 1). We hypothesized that this might be due to increased sensitivity of our NHEJ-deficient parent strain to phleomycin, which induces double-stranded DNA breaks. However, the use of a Cas9-expressing parent strain with intact *YKU80* did not improve transformant yield to the level of a *NAT* control (Table S1). Reducing the phleomycin selection concentration from 200 to 100 μg/mL yielded only a modest (~2-fold) increase in the number of transformants obtained (Table S2). These data suggest that, while *BLE* can effectively select for transformants, it does so with lower efficiency than other markers under these conditions.

In summary, we have expanded the number of dominant selection methods available for *C. neoformans* to seven, including three well-established selection drugs (hygromycin B, G418, and nourseothricin), two dominant prototrophic methods (acetamide and phosphite prototrophy), one newly established selection drug (blasticidin S), and one previously reported yet unutilized selection drug (phleomycin). We have adapted these to be compatible with marker-guide fusion DNA constructs that we have recently demonstrated to enhance homology-dependent genome-editing efficiency, especially when combined with a reversible Ku80 mutation. With the rapidly expanding genetic toolbox for *C. neoformans*, the availability of multiple robust and validated selection methods will provide significant flexibility in constructing strains incorporating multiple sequential modifications.

## SUPPLEMENTAL MATERIAL

Fig. S1 spectrum.01935-25-s0001.tif Drug concentration titration.

Fig. S2 spectrum.01935-25-s0002.tif Insets of colony photographs.

Supplemental material spectrum.01935-25-s0003.docx Supplemental materials and methods; supplemental figure legends; Tables S1 and S2.

Table S3 spectrum.01935-25-s0004.xlsx Primers and plasmids.

ASM does not own the copyrights to Supplemental Material that may be linked to, or accessed through, an article. The authors have granted ASM a non-exclusive, world-wide license to publish the Supplemental Material files. Please contact the corresponding author directly for reuse.
