# Supplementary figures and images for: CRISPR/Cas9-compatible plasmids enabling seven dominant genetic selection methods for the human fungal pathogen Cryptococcus neoformans
Source: Microbiol Spectr. 2025 Sep 25;13(11):e01935-25. doi: 10.1128/spectrum.01935-25 (PMC12584689; doi:10.1128/spectrum.01935-25)

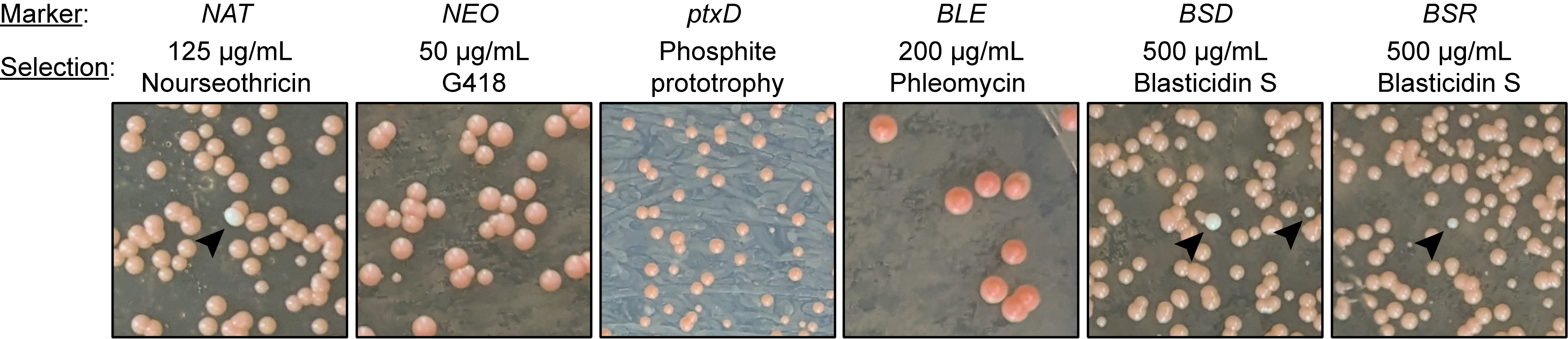

Supplement: Fig. S2 — Insets of colony photographs. [file spectrum.01935-25-s0002.tif]
