# Supplementary material for: CRISPR/Cas9-compatible plasmids enabling seven dominant genetic selection methods for the human fungal pathogen Cryptococcus neoformans
Source: Microbiol Spectr. 2025 Sep 25;13(11):e01935-25. doi: 10.1128/spectrum.01935-25 (PMC12584689; doi:10.1128/spectrum.01935-25)
Supplement: Supplemental material — s and methods; supplemental figure legends; Tables S1 and S2. [file spectrum.01935-25-s0003.docx]

**SUPPLEMENTAL MATERIAL**

**CRISPR/Cas9-compatible plasmids enabling seven dominant genetic selection methods for the human fungal pathogen *Cryptococcus neoformans***

Michael J. Boucher^1^, Hiten D. Madhani^1,*^

^1^Department of Biochemistry and Biophysics, University of California, San Francisco, CA 94158, USA

*Correspondence: hitenmadhani@gmail.com

**SUPPLEMENTAL MATERIALS AND METHODS**

**Yeast strains and growth conditions**

*C. neoformans* strains used in this study are listed in Table S3. Standard yeast culture was performed at 30°C in YPAD growth medium (1% yeast extract, 2% peptone, 2% glucose, 150 mg/L L-tryptophan, 40 mg/L adenine). Solid media contained 2% agar. Liquid culture was performed on an orbital shaker (200 rpm) or roller drum. Yeast transformed with drug resistance markers were selected on YPAD agar containing 125 μg/mL nourseothricin sulfate (Jena Bioscience AB-102XL), 50 μg/mL G418 sulfate (Corning 61-234-RG), 100-200 μg/mL phleomycin (Invivogen ant-ph-5p), or 500 μg/mL blasticidin S HCl (Goldbio B-800-1). Selection of phosphite prototrophs was performed as previously described (1)  on synthetic complete (SC) agar containing phosphate-free yeast nitrogen base (ForMedium CYN6701) supplemented with 7.35 mM phosphite (Sigma 04283), 22 mg/L uracil, 21 mg/L adenine, 8.6 mg/L para-aminobenzoic acid, 174 mg/L L-leucine, 80 mg/L L-tryptophan, and 85 mg/L of each L-alanine, L-arginine-HCl, L-asparagine, L-aspartic acid, L-cysteine-HCl, L-glutamine, L-glutamic acid, glycine, myo-inositol, L-isoleucine, L-methionine, L-phenylalanine, L-proline, L-serine, L-threonine, L-tyrosine, L-valine, L-histidine-HCl, and L-lysine-HCl.

**Molecular cloning**

Molecular cloning was performed using HiFi DNA Assembly Master Mix (NEB E2521L). Primers and plasmids used in this study are listed in Table S3. *C. neoformans* codon-optimized *BLE*, *BSD*, *BSR*, and *ptxD* markers containing an intron were designed using a previously reported schema (2) and synthesized by Twist Bioscience. The backbone of plasmid pSDMA25 (lacking its *NAT* marker) (3) was amplified with primers MJB1039 and MJB1040. Synthesized markers were amplified with primers MJB1041 and MJB1042 (*BLE*), MJB1043 and MJB1044 (*BSD*), MJB1045 and MJB1046 (*BSR*), or MJB1047 and MJB1048 (*ptxD*) and cloned into the pSDMA25 backbone. To expand the family of fused marker-guide plasmids (4), markers were amplified from safe haven 1 plasmids with MJB1065 and MJB1066, which anneal in the *ACT1* promoter and *TRP1* terminator, respectively, and were cloned into SacI/SpeI-digested pBHM2616.

**Fused marker-guide constructs**

PCR generation of fused marker-guide constructs was performed using ExTaq polymerase (Takara RR001B) as previously described (4). Briefly, marker fragments containing an overhang corresponding to a 20 bp *ADE2* guide sequence were amplified from pBHM2617, pBHM2618, pBHM2678, pBHM2679, pBHM2680, or pBHM2681 with primers MJB537 and ADE2-1_PCnU6_R. Guide fragments containing the same *ADE2* guide sequence were amplified from pBHM2616 with primers ADE2-1_sgRNA_F and MN0504. Equal volumes of separate marker and guide PCR products were combined, purified on a PCR cleanup column (Machery-Nagel), and eluted in 40 μL of water. Fragments were fused in a second round of PCR (10 cycles) using 10 μL of the eluted marker-guide fragments as template without primers. Fused marker-guide constructs were then produced by 30 cycles of nested PCR with primers containing 50 bp homology arms flanking the *ADE2* coding sequence (MJB1069 and MJB1070) using 1 uL of the fused product as template. Products were then purified on a PCR cleanup column and eluted with water.

**Yeast transformation**

Yeast transformation was performed as previously described (2, 3). Briefly, overnight cultures grown in YPAD were diluted to an OD_600_ of 0.2 in 100 mL YPAD and cultured 4-5 hr to an OD_600_ of 0.7-0.9. Yeast were pelleted at 3000 x*g* at 4°C and washed twice with 25 mL of cold water. Pellets were resuspended in 10 mL electroporation buffer (10 mM Tris-HCl, pH 7.4, 1 mM MgCl_2_, 270 mM sucrose) with 1 mM DTT and incubated on ice for 1 hr. Yeast were pelleted at 3000 x*g* at 4°C, and 250 μL cold electroporation buffer (without DTT) was added, yielding approximately 500 μL of competent cells. For each transformation 50 μL of cells were mixed with 1 μg fused marker-guide PCR product, transferred to a prechilled 0.2 cm electroporation cuvette, and electroporated at 500 V, 400 Ω, and 250 μF on a BTX Gemini X2 electroporator. Yeast were resuspended in 1 mL YPAD and incubated rotating at 30°C for 2 hr. To calculate the number of transformants obtained per electroporation, dilutions corresponding to 1%, 10%, and 70% of the total electroporation volume were plated onto selective media and incubated at 30°C. Colony forming units (CFUs) were enumerated after 3 (all markers except *ptxD*) or 4 (*ptxD*) days of growth. To ensure use of clonal isolates in subsequent genotyping steps (see below), single colonies were re-streaked onto selective agar and grown at 30°C before culture for genomic DNA (gDNA) isolation.

**gDNA isolation and transformant genotyping**

Single colonies re-streaked from transformation plates were cultured in 4-5 mL liquid YPAD overnight in the absence of selection at 30°C. The next day, cultures were harvested by centrifugation, washed in water, and stored at -20°C until further processing.

To isolate gDNA, cell pellets were resuspended in 500 μL lysis buffer (100 mM Tris-HCl, pH 8.0, 50 mM EDTA, 1% SDS) and transferred to a 2 mL screw-cap tube containing ~200 μL 0.5 mm zirconia/silica beads (BioSpec 11079105Z). Samples were vortexed on “high” on a Vortex-Genie 2 with an attached microcentrifuge tube insert for 5 min at room temperature and were pelleted at 21,000 x*g* for 5 min at 4°C. Supernatants were transferred to fresh tubes, and 200 μL 10 M ammonium acetate was added. Samples were incubated at 65°C for 5 min followed by -20°C for 5 min, and 500 μL chloroform was added. Samples were mixed by inversion and centrifuged at 21,000 x*g* for 5 min at 4°C. The aqueous phase was transferred to a fresh tube containing 500 μL isopropanol, and samples were mixed by inversion and centrifuged at 21,000 x*g* for 20 min at 4°C. The supernatant was removed, and the pellet was washed with 700 μL cold 70% ethanol followed by centrifugation for 5 min at 21,000 x*g* at 4°C. The supernatant was removed, and pellets were air-dried and resuspended in 100 μL water.

Transformants were genotyped by diagnostic PCR using primers outlined in Fig. 2B and Table S3 to detect 1) modified *ADE2* upstream knockout junctions; 2) modified *ADE2* downstream knockout junctions; 3) internal *ADE2* coding sequence present only in unmodified strains; or 4) the safe haven 1 locus (positive control for successful gDNA isolation). PCR was performed using a previously described colony PCR protocol (2), except that 1 μL of isolated gDNA was used as template in place of microwaved colonies, as purified gDNA is expected to yield fewer false negative results.

**SUPPLEMENTAL FIGURES AND LEGENDS**

**
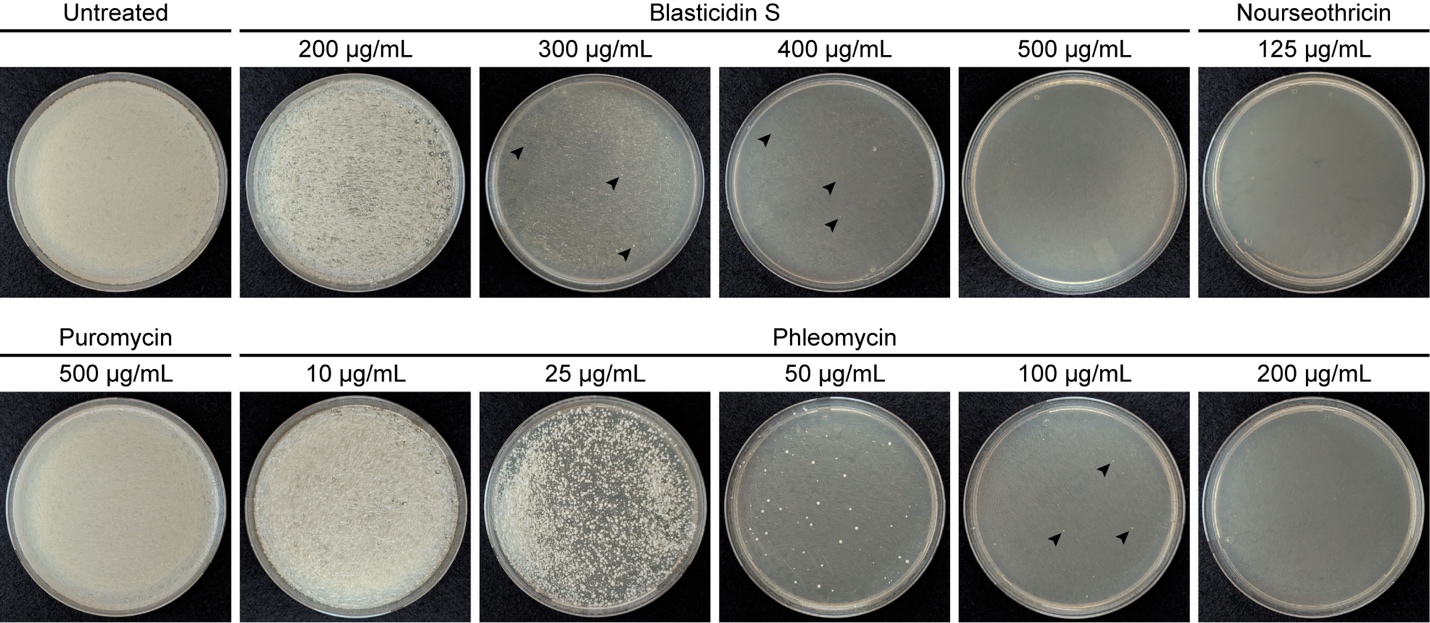
**

**Figure S1. Identification of selection drugs that inhibit *C. neoformans* growth.** 5 x 10^7^ CFUs of *C. neoformans* KN99α (CM026) were spread onto YPAD agar plates containing drugs of interest at the indicated concentrations and were incubated at 30°C for 3 days before imaging. Small background colonies are indicated with black arrowheads.


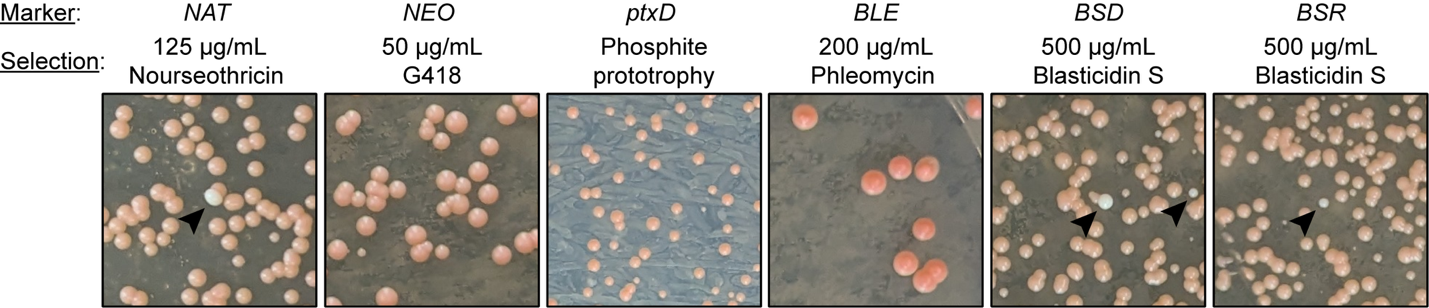


**Figure S2. Additional data supporting efficient disruption of *ADE2* using novel and established selectable markers.** Images are insets of plates displayed in Fig. 2A to highlight the pink/red colony color characteristic of *ade2*Δ yeast. For comparison, black arrowheads indicate rare white colonies in which *ADE2* was not successfully disrupted.

**SUPPLEMENTAL TABLES**

**Table S1. Transformants obtained in a Cas9-expressing, *YKU80*-intact background.**

| **Marker** | **Selection** | **Experiment number** | **Transformants** | **Percentage of transformants relative to *NAT*** |
| --- | --- | --- | --- | --- |
| *NAT* | Nourseothricin (125 μg/mL) | 1 | 23,200 | 100.0 |
|  |  | 2 | 11,900 | 100.0 |
| *BLE* | Phleomycin (200 μg/mL) | 1 | 2900 | 12.5 |
|  |  | 2 | 2100 | 17.6 |

**Table S2. Transformants obtained in a Cas9-expressing, *yku80*-blaster background with reduced phleomycin concentrations.**

| **Marker** | **Selection** | **Experiment number** | **Transformants** | **Percentage of transformants relative to *NAT*** |
| --- | --- | --- | --- | --- |
| *NAT* | Nourseothricin (125 μg/mL) | 1 | 1700 | 100.0 |
|  |  | 2 | 630 | 100.0 |
|  |  | 3 | 1010 | 100.0 |
| *BLE* | Phleomycin (200 μg/mL) | 1 | 250 | 14.7 |
|  |  | 2 | 63 | 10.0 |
|  |  | 3 | 106 | 10.5 |
| *BLE* | Phleomycin (150 μg/mL) | 1 | 280 | 16.5 |
|  |  | 2 | 120 | 19.0 |
|  |  | 3 | 191 | 18.9 |
| *BLE* | Phleomycin (100 μg/mL) | 1 | 420 | 24.7 |
|  |  | 2 | 166 | 26.3 |
|  |  | 3 | 250 | 24.8 |

**SUPPLEMENTAL REFERENCES**

1. Khongthongdam M, Phetruen T, Chanarat S. 2024. Development of *ptxD*/Phi as a new dominant selection system for genetic manipulation in *Cryptococcus neoformans*. Microbiol Spectr 13:e01618-24.
2. Huang MY, Joshi MB, Boucher MJ, Lee S, Loza LC, Gaylord EA, Doering TL, Madhani HD. 2021. Short homology-directed repair using optimized Cas9 in the pathogen *Cryptococcus neoformans* enables rapid gene deletion and tagging. Genetics 220:iyab180.
3. Arras SDM, Chitty JL, Blake KL, Schulz BL, Fraser JA. 2015. A genomic safe haven for mutant complementation in *Cryptococcus neoformans*. PLOS ONE 10:e0122916.
4. Nalley MJ, Banerjee S, Huang MY, Madhani HD. 2025. Near 100% efficient homology-dependent genome engineering in the human fungal pathogen *Cryptococcus neoformans*. G3 jkaf118.
